# Multilevel Faith-Based Public Health Initiative in Rural Alabama, 2017

**DOI:** 10.5888/pcd16.190057

**Published:** 2019-08-29

**Authors:** Alicia R. Powers, Ruth W. Brock, Katie Funderburk, Sondra M. Parmer, Barb Struempler

**Affiliations:** 1 Hunger Solutions Institute, Auburn University, Auburn, Alabama; 2 Alabama High Obesity Program, Alabama Cooperative Extension System, Auburn University, Auburn, Alabama; 3 Supplemental Nutrition Assistance Program–Education, Alabama Cooperative Extension System, Auburn University, Auburn, Alabama; 4 Alabama Cooperative Extension System, Auburn University, Auburn, Alabama

## Abstract

Recent shifts in public health approaches to reduce and prevent chronic disease encourage interventions to include multiple levels of the social ecological model. The objective of this 1-group pretest–posttest study was to determine differences in faith community policies and environments; interpersonal support; and individual behavior before and after Live Well Faith Communities, a 9-week, faith-based health promotion initiative. The study included a convenience sample of faith communities and participants. Validated instruments assessed faith communities’ policies and environments and participants’ interpersonal and individual practices and behaviors. Seventy-two small-group sessions with 737 adults were implemented in 14 faith communities. Faith communities adopted policies requiring healthy options for meals and snacks and implemented environmental changes to promote healthy eating and physical activity. Participants reported significant improvements in healthy eating encouragement, shopping practices, and vegetable consumption. Multilevel interventions prompt community organizations to become healthier places and individuals to adopt healthy lifestyles.

**Summary**

**What is already known on this topic?**

Recent shifts in public health approaches to reduce and prevent obesity focus on multilevel interventions encompassing organizational, interpersonal, and individual changes as emphasized in the social ecological model.

**What is added by this report?**

This report operationalizes a multilevel, faith-based health promotion initiative and provides evidence of such an initiative on multiple levels of the social ecological model.

**What are the implications for public health practice?**

Public health practitioners should prioritize partnerships with community organizations who can influence multiple levels of the social ecological model to provide support for healthy behaviors in at-risk populations.

## Introduction

Overweight and obesity are national epidemics affecting more than two-thirds of adults in the United States (1); the Southeast has higher obesity rates than most other regions (2). Alabama ranks fifth nationally in obesity prevalence; 36.3% of adults in Alabama are obese (3).

Racial/ethnic minority populations tend to have higher rates of obesity than the non-Hispanic white population. Almost half (48.1%) of non-Hispanic black adults and 42.5% of Hispanic adults are obese, compared with 34.5% of non-Hispanic white adults (4). Many studies also indicate a correlation between obesity and socioeconomic status and education level. Not only is obesity a public health issue itself but obesity leads to other health problems, such as cardiovascular disease, cancer, diabetes, respiratory disorders, and more.

Recent shifts in public health approaches to reduce and prevent obesity and chronic diseases expand the focus from individual-level behavior change interventions to multilevel interventions encompassing policy changes, cultural shifts, environmental changes, interpersonal influence, and individual-level behavior changes as emphasized in the social ecological model. Because of this shift to multilevel interventions, community organizations, such as schools, workplaces, and faith communities, are increasingly common settings for health promotion initiatives. Growing evidence supports the effectiveness of faith-based health promotion initiatives (5–8).

## Purpose and Objective

In 2017, the Alabama Cooperative Extension System at Auburn University (Extension) launched Live Well Faith Communities (LWFC), a 9-week, multilevel, faith-based health promotion initiative. The objective of this 1-group pretest–posttest study was to determine differences in faith community (institutional) policies, environments, and programs; interpersonal support; and faith community member (individual) behavior before and after participating in LWFC. We sought to determine differences in participating faith communities’ health infrastructure, partnerships, and programs; healthy eating policies, environments and programs; and physical activity policies, environments, and programs. Additionally, we sought to determine differences in perceived social support and behaviors related to healthy eating and physical activity of faith community members participating in LWFC.

## Intervention Approach

The social ecological model recognizes and emphasizes the interaction among multiple factors influencing a person’s behavior. This model consists of 5 levels of influence for health-related behaviors: individual, interpersonal, institutional, community, and public policy (9). Given the setting of LWFC, researchers used institutional, interpersonal, and individual levels for development, implementation, and evaluation of LWFC.

LWFC integrated the institutional level of the social ecological model through Extension personnel who 1) supported faith community leaders in conducting a needs assessment, 2) provided technical assistance, and 3) consulted with members of the faith community to inform, initiate, expand and/or sustain policy, systems, and environmental (PSE) strategies. PSE strategies suggested in the LWFC protocol included planning healthy meals to serve at faith community events; partnering with local farmers to sell low-cost produce at faith community facilities; developing a policy requiring fruits, vegetables, and/or water be served at any faith community gathering where food and/or beverages are served; and starting a walking or exercise group.

For the interpersonal level of the social ecological model, LWFC components were the small group environment of direct education lessons and a faith community champion. Extension personnel partnered with each participating faith community to identify a faith community member to serve as the champion. This person was the liaison between Extension personnel and the faith community. In addition to supporting the planning, publicizing, and facilitating logistics of LWFC, this person also supported participants as they practiced principles learned in the direct education lessons and faith community leaders as they implemented evidence-based PSE strategies.

Extension personnel conducted 9 weekly small-group direct education lessons focused on positively influencing individual healthy eating and physical activity behaviors. These 9-week programs were conducted on a rolling basis throughout 2017. Lessons topics included eating smart at home; planning, shopping, preparing, and choosing healthy foods; making smart drink choices; and moving more throughout the day. A protocol and curriculum were provided to all Extension personnel. These materials included the following: a lesson overview, a detailed lesson plan, a handout for participants, a recipe for demonstration and tasting, PowerPoint slides with script, discussion questions, sample physical activities, social media posts, and a PSE strategy for discussion. Materials for the direct education portion of LWFC were adapted from *Faithful Families Eating Smart and Moving More* (10).

## Evaluation Methods

A 1-group pretest–posttest study design assessed institutional healthy eating and physical activity policies, environments, and programs; interpersonal social support for healthy eating and physical activity; and individual healthy eating and physical activity behavior before and after participation in LWFC. The evaluations, like the 9-week sessions, were conducted on a rolling basis throughout 2017. The study protocol was approved by the Auburn University Institutional Review Board.

### Sample

Trained Extension personnel recruited faith communities to participate in LWFC. Although these Extension personnel serve all counties in Alabama, we prioritized 14 counties with adult obesity rates greater than 40%. Supplemental Nutrition Assistance Program–Education (SNAP-Ed) personnel focused recruitment on faith communities in the same zip code area as a SNAP-Ed qualifying school, defined as school in which 50% or more of students receive a free or reduced-price school meal. Expanded Food and Nutrition Education Program educators focused recruitment on faith communities in which at least 75% of participants were low-income adults with children living at home, low-income pregnant teenagers or adults, or low-income grandparents who provide primary care for grandchildren.

Researchers trained and provided information and support to Extension personnel on faith community recruitment and partnership procedures. Extension personnel consulted an information sheet and an agreement of roles and responsibilities during an in-person, email, or telephone conversation to recruit potential faith communities. When faith communities agreed to partner with Extension to implement LWFC, Extension personnel and faith community leadership completed and submitted to researchers the written agreement on roles and responsibilities.

To recruit participants in LWFC, researchers developed and provided a poster and bulletin/newsletter insert for Extension personnel to provide to the faith community for publicizing LWFC and its start date. LWFC was offered to faith community members as well as members of the surrounding community.

We used a convenience sample of faith communities and adults participating in LWFC for this study. All participants aged 18 or younger were excluded from analysis.

### Surveys

We developed a faith community assessment as a pretest and posttest to assess the institutional level of the social ecological model. We adapted this survey from the Faithful Families Faith Communities Assessment (10), Live Well Greenville House of Worship Assessment (Meghan M. Slining, PhD, MPH, Furman University, LiveWell Greenville; verbal, electronic, and written communication, 2016), and the Texas A&M Capacity and Readiness Church Health Assessment (11). We conducted this assessment among each faith community’s leadership, which included the faith community leader, the faith community champion, the health ministry team leader, and/or the health ministry team members. Twelve questions focused on general information about the faith community and its membership. Five questions determined the faith community’s infrastructure related to health programming, such as a health ministry team, leader, and budget. Twenty questions focused on physical activity policies, environments, and programs. These questions emphasized physical activity opportunities made available by the faith community, such as an indoor gym, walking trail, playground, group exercise classes, walking clubs, or sports teams as well as promotion of physical activity in printed materials and policies. Thirty-two questions focused on healthy eating policies, environments, and programs. These questions emphasized guidelines requiring certain foods at faith community meals or snacks, food preparation, food service equipment, group classes on healthy eating, and promotion of healthy eating in faith community printed materials and policies.

We developed a participant assessment as a pretest and posttest to assess interpersonal and individual levels of the social ecological model. The assessment was developed from previously validated instruments (12–15).

To measure interpersonal support related to healthy eating and physical activity, the participant assessment included 10 questions from the Social Support and Eating Habits Survey (15) and 10 questions from the Social Support and Exercise Survey (15). These instruments measured 3 areas of social support: healthy eating encouragement, healthy eating discouragement, and physical activity participation encouragement. We used validated scoring procedures for the social support scales for analyses (15).

For the individual level of the social ecological model, the participant assessment measured practices and behaviors in food resource management, food safety, food purchasing, healthy eating, and physical activity (12–14).

### Procedures

After recruitment and commitment of faith communities in early 2017, Extension personnel engaged faith community leadership to implement LWFC. The initial step included identifying and training a faith community member to serve as the LWFC faith community champion, which was integral to influencing the interpersonal level of the social ecological model in LWFC.

Next, Extension personnel helped the faith community leader, the faith community champion, and the health ministry team (if appropriate) complete the faith community assessment pretest. Extension personnel provided technical assistance to faith community leadership to promote use of assessment findings in developing a 9-week action plan. The action plan detailed activities necessary to initiate, expand, and/or sustain PSE strategies in the faith community. During the 9 weeks, Extension personnel provided technical assistance and consultation for implementation of the action plan.

Simultaneously, Extension personnel, in partnership with the faith community champion, helped participants complete the paper-and-pencil pretest during the first weekly small-group direct education lesson. Extension personnel and faith community champions also jointly implemented each of the small-group direct education lessons using the LWFC protocol and curriculum. The integration of the faith community champion into the program and the use of small groups in weekly sessions demonstrated the interpersonal level of the social ecological model in LWFC. The intent to positively influence individual healthy eating and physical activity behaviors further demonstrated the inclusion of the individual level of the social ecological model.

At the last weekly small-group direct education lesson, Extension personnel and faith community champions helped participants complete the posttest. At the conclusion of the final lesson, Extension personnel supported faith community leadership in completing the faith community assessment posttest.

We analyzed survey data by using SPSS version 24 (IBM Corporation) for Windows. Researchers used descriptive statistics to determine means and percentages for demographic information. We used the Mann–Whitney *U* test and the independent-samples *t* test to assess differences in respondents who completed the pretest and respondents who completed the posttest. We considered a *P* value of <.05 significant.

## Results

Sixteen Extension personnel implemented LWFC in 14 faith communities in 8 rural counties with adult obesity rates greater than 40%. Faith communities implemented 11 PSE strategies. Of 8 faith communities adopting guidelines requiring healthy options at meals or snacks, 2 required fruits, 3 required vegetables, 2 required nonfried foods, and 1 required low-sugar or no-sugar-added foods. One faith community created an onsite garden, one began providing physical activity opportunities at meetings or functions, and one began offering group exercise classes.

Extension personnel provided 72 direct education classes for 737 adults; 119 adult participants completed the participant assessment pretest (n = 79) and/or posttest (n = 48).

The average survey respondent was a middle-aged (mean age, 57.5 y), non-Hispanic black woman. Most (84%) respondents had at least a high school diploma or equivalent (Table 1).

**Table 1:** Demographic Characteristics of Adults (N = 119) Completing the Participant Pretest (n = 79) and/or Posttest (n = 48) for Live Well Faith Communities, a 9-Week, Multilevel Faith-Based Health Promotion Initiative, Alabama, 2017^a^

Demographic Characteristic	No. of Participants Who Answered Question	Value^b^
**Age, mean (SD), y**	58	57.5 (14.4)
**Sex**		
Male	60	16 (27)
Female		44 (73)
**Hispanic/Latino**	52	52 (100)
**Race**		
American Indian or Alaskan Native	59	1 (2)
Black or African American		58 (98)
**Education**		
Some high school	60	10 (17)
Graduated from high school or has GED		13 (22)
Some college		21 (35)
Graduated from college		16 (27)
**Marital status**		
Married	55	33 (60)
Single		22 (40)

a The Alabama Cooperative Extension System at Auburn University launched Live Well Faith Communities, a 9-week, multilevel, faith-based health promotion initiative in 14 faith communities in 8 counties in which the prevalence of obesity was >40%.

b All values are number (percentage) unless otherwise indicated. Not all participants answered all questions. Percentages may not add to 100 because of rounding.

At the interpersonal level, the mean (SD) score for healthy eating encouragement improved significantly (*t*_109_ = −4.87; *P* < .001) among respondents from 5.6 (4.2) on the pretest to 9.6 (4.2) on the posttest (Table 2). Healthy eating discouragement and physical activity encouragement did not differ significantly from pretest to posttest.

**Table 2:** Interpersonal and Individual-Level Variables Among Participants Completing the Pretest and/or Posttest in Live Well Faith Communities, Alabama, 2017^a^

Variable	Pretest (n = 79)^b^	Posttest (n = 48)^b^	Test Statistic (*P* Value)^c^
**Interpersonal^d^, mean (SD)**			
Healthy eating encouragement	5.6 (4.2)	9.6 (4.2)	*t*_109_ = −4.87 (<.001)
Healthy eating discouragement	13.7 (4.5)	14.3 (4.0)	*t*_107_ = −0.65 (.52)
Physical activity encouragement	13.4 (10.2)	15.9 (11.3)	*t*_104_ = −1.16 (.25)
**Individual**			
**Food resource management^e^**			
Plan meals ahead of time	28 of 79 (35.4%)	20 of 43 (46.5%)	*U* = 1,875.0 (.31)
Think about healthy foods when planning for their family	30 of 78 (38.5%)	30 of 43 (69.8%)	*U* = 2,259.5 (.001)
Shop with a grocery list	32 of 77 (41.6%)	19 of 43 (44.2%)	*U* = 1,779.0 (.49)
Compare prices before buying	42 of 78 (53.8%)	30 of 42 (71.4%)	*U* = 1,988.0 (.045)
Use nutrition facts to make food choices	20 of 79 (25.3%)	18 of 43 (41.9%)	*U* = 2,144.0 (.01)
**Food safety** f			
Let meat or dairy food sit out	57 of 76 (75.0%)	29 of 42 (69.0%)	*U* = 1,584.0 (.94)
Thaw frozen foods at room temperature	25 of 75 (33.3%)	17 of 42 (40.5%)	*U* = 1,665.5 (.59)
**Food purchasing choices^g^**			
Buy low-fat or fat-free milk or dairy foods	29 of 79 (36.7%)	23 of 43 (53.5%)	*U* = 2,048.5 (.054)
Buy food with lower added sugar	25 of 79 (31.6%)	21 of 43 (48.8%)	*U* = 2,112.0 (.02)
Buy food with low salt	23 of 77 (29.9%)	18 of 43 (41.9%)	*U* = 1,952.5 (.09)
**Healthy eating and physical activity practices^h^**			
Average daily vegetable consumption, no. (mean), cups	1.5 (0.8)	1.8 (0.6)	*t*_119_ = −2.50 (.01)
Average daily fruit consumption, no. (mean), cups	1.4 (0.9)	1.6 (0.9)	*t*_118_ = −0.73 (.46)
Average exercise per week, no. (mean), days	2.3 (1.6)	2.5 (1.6)	*t*_116_ = −0.45 (.66)

a The Alabama Cooperative Extension System at Auburn University launched Live Well Faith Communities, a 9-week, multilevel, faith-based health promotion initiative in 14 faith communities in 8 counties in which the prevalence of obesity was >40%.

b The largest number of participants completing any question on the pretest was 79, and the largest number of participants completing any question on the posttest was 48. Some participants completed only the pretest, some completed only the posttest, and some completed both pretest and posttest.

c Independent samples *t* test determined significance for differences in mean (SD) between pretest and posttest, and independent samples Mann–Whitney *U* test determined significance for differences in percentage between pretest and posttest.

d Based on Social Support and Eating Habits Survey (15) and the Social Support and Exercise Survey (15). Scale for healthy eating encouragement ranges from 5 to 25, with higher scores indicating greater encouragement. Scale for healthy eating discouragement ranges from 5 to 25, with higher scores indicating greater discouragement. Scale for physical activity encouragement ranges from 11 to 55, with higher scores indicating greater encouragement.

e Based on University of California Cooperative Extension’s Plan, Shop, Save and Cook Survey (12) and Cooking Matters for Adults Survey (13). Percentage of respondents who answered “often” or “always.”

f Based on The Expanded Food and Nutrition Education Program’s Behavior Checkist (14). Percentage of respondents who answered “often” or “always.”

g Based on Cooking Matters for Adults Survey (13) and SNAP-Ed Evaluation Framework and Interpretive Guide (16). Percentage of respondents who answered “often” or “always.”

h Based on SNAP-Ed Evaluation Framework and Interpretive Guide (16).

At the individual level in food resource management, responses differed significantly from pretest to posttest in 3 areas. At pretest, 38.5% of respondents indicated they often or always think about healthy food choices when planning foods for their family, whereas at posttest, 69.8% of respondents indicated this (*U* = 2,259.5; *P* = .001). At pretest, 53.8% of respondents indicated they often or always compare prices before buying foods, whereas at posttest, 71.4% indicated this (*U* = 1,988.0 *P* = .045), and at pretest, 25.3% indicated they often or always use nutrition facts to make food choices, whereas at posttest, 41.9% indicated this (*U* = 2,144.0; *P* = .01).

Also at the individual level, in food purchasing choices, 31.6% of pretest respondents indicated they often or always purchase foods with lower added sugar, whereas 48.8% indicated this at posttest (*U* = 2,112.0; *P* = .02). Finally, the average daily vegetable consumption among respondents differed significantly from pretest (1.5 [SD, 0.8] cups) to posttest (1.8 [SD, 0.6] cups) (*t*_119_ =−2.50; *P* = .01). 

## Implications for Public Health

LWFC supported 14 faith communities in rural Alabama in becoming healthier places and 737 adults in adopting healthier lifestyles. The initiative positively influenced 3 levels of the social ecological model: institutional, interpersonal, and individual. At the institutional level, faith communities shifted policies and created environments to foster healthy eating and physical activity in the faith community setting. As hypothesized, the traditional role of the faith community in supporting positive development of its members, the intentional inclusion of small-group direct education classes, and the partnership with the faith community champion bolstered social support for participants in LWFC, which resulted in participants recognizing greater support for healthy eating. Furthermore, we noted key behavioral changes, including improved practices in making healthy choices and improved healthy eating behaviors.

Our study has several limitations. First, the research design, a 1-group pretest–posttest, lacked a comparison group, which is necessary for determining whether changes among participants resulted from participation in LWFC. Second, data were self-reported, and self-reported data are subject to such biases as recall bias and social desirability bias. Third, the convenience sampling method and homogenous sample limit generalizability of the study’s findings.

Although these methodologic factors may have introduced limitations, they also were key strengths to our study. Convenience sampling was necessary because of the community-engaged approach of this initiative. Partnership with the faith community promoted adoption of PSE changes and recruitment of faith community members. Furthermore, the faith community assessment instrument was intentionally designed as a self-assessment, so that it would support discussion and contemplation of potential PSE strategies appropriate at the faith community (institutional) level. Although the study’s generalizability is limited because of the homogeneity of the sample, the study provides evidence of the effectiveness of a multilevel, faith-based health promotion initiative in an African American population in the Southeast, which is at greater risk for obesity and chronic diseases than other populations. Our study suggests that faith communities are promising settings for public health initiatives aiming to influence multiple levels of the social ecological model.

## References

[1] National Center for Health Statistics. Health, United States, 2017: with special feature on mortality. Hyattsville (MD): US Government Printing Office; 2018. 30702833

[2] Centers for Disease Control and Prevention. Estimated county-level prevalence of diabetes and obesity — United States, 2007. MMWR Morb Mortal Wkly Rep 2009;58(45):1259–63. 19940830

[3] Segal LM , Rayburn J , Beck S . The state of obesity 2017: better policies for a healthier America. https://www.stateofobesity.org. Accessed May 9, 2019.

[4] Ogden CL , Carroll MD , Fryar CD , Flegal KM . Prevalence of obesity among adults and youth: United States, 2011–2014. Hyattsville (MD): National Center for Health Statistics; 2015 Nov. DHHS Publication No. 2016-1209.

[5] Bopp M , Fallon EA . Health and wellness programming in faith-based organizations: a description of a nationwide sample. Health Promot Pract 2013;14(1):122–31. 10.1177/1524839912446478 23008281

[6] Peterson J , Atwood JR , Yates B . Key elements for church-based health promotion programs: outcome-based literature review. Public Health Nurs 2002;19(6):401–11. 10.1046/j.1525-1446.2002.19602.x 12406175

[7] Campbell MK , Hudson MA , Resnicow K , Blakeney N , Paxton A , Baskin M . Church-based health promotion interventions: evidence and lessons learned. Annu Rev Public Health 2007;28(1):213–34. 10.1146/annurev.publhealth.28.021406.144016 17155879

[8] DeHaven MJ , Hunter IB , Wilder L , Walton JW , Berry J . Health programs in faith-based organizations: are they effective? Am J Public Health 2004;94(6):1030–6. 10.2105/AJPH.94.6.1030 15249311 PMC1448385

[9] National Cancer Institute. Theory at a glance: a guide for health promotion practice. Washington (DC): National Institutes of Health; 2005.

[10] Dunn C , Hardison-Moody A , Jones L , Rhew L , Thomas C . Faithful families eating smart and moving more. Raleigh (NC): North Carolina State University; 2016.

[11] Motley M . Identifying and exploring capacity and readiness of faith-based organizations implementing lifestyle-related chronic disease health programs [dissertation]. Blacksburg (VA): Virginia Polytechnic Institute and State University; 2015.

[12] Kaiser L , Schneider C , Chaidez V , Neelon M , Algert S , Smith D , Plan, shop, save, cook: influence of SNAP on program outcomes. J Nutr Educ Behav 2013;45(4S):S16–7. 10.1016/j.jneb.2013.04.045

[13] Pinard CA , Uvena LM , Quam JB , Smith TM , Yaroch AL . Development and testing of a revised cooking matters for adults survey. Am J Health Behav 2015;39(6):866–73. 10.5993/AJHB.39.6.14 26450554

[14] US Department of Agriculture, National Institute of Food and Agriculture. EFNEP behavior checklist review. Washington (DC): US Department of Agriculture; 2012.

[15] Sallis JF , Grossman RM , Pinski RB , Patterson TL , Nader PR . The development of scales to measure social support for diet and exercise behaviors. Prev Med 1987;16(6):825–36. 10.1016/0091-7435(87)90022-3 3432232

[16] US Department of Agriculture. SNAP-Ed evaluation and interpretive guide. https://snaped.fns.usda.gov/program-administration/snap-ed-evaluation-framework. Accessed June 10, 2019.

